# New Tardigrade Opsins and Differential Expression Analyses Show Ontogenic Variation in Light Perception

**DOI:** 10.1093/gbe/evab164

**Published:** 2021-07-13

**Authors:** James F Fleming, Davide Pisani, Kazuharu Arakawa

**Affiliations:** 1 Keio University Institute for Advanced Biosciences, Tsuruoka City, Yamagata, Japan; 2 University of Oslo Natural History Museum, Oslo, Norway; 3 University of Bristol, Bristol Life Sciences Building, Bristol, United Kingdom

**Keywords:** Tardigrada, opsin, phylogeny, transcriptomics, Ecdysozoa

## Abstract

Opsins are light-sensitive proteins involved in many photoreceptive processes, including, but not limited to, vision and regulation of circadian rhythms. Arthropod (e.g., insects, spiders, centipedes, and crabs) opsins have been extensively researched, but the relationships and function of opsins found in lineages that are evolutionarily closely related to the arthropods remains unclear. Multiple, independent, opsin duplications are known in Tardigrada (the water bears), evidencing that protostome opsin duplications are not limited to the Arthropoda. However, the relationships, function, and expression of these new opsins are still unknown. Here, we use two tardigrade transcriptomes with deep coverage to greatly expand our knowledge of the diversity of tardigrade opsins. We reconstruct the phylogenetic relationships of the tardigrade opsins and investigate their ontogenetic expression. We found that while tardigrades have multiple opsins that evolved from lineage-specific duplications of well-understood arthropod opsins, their expression levels change during ontogeny such that most of these opsins are not co-temporally expressed. Co-temporal expression of multiple opsins underpins color vision in Arthropoda and Vertebrata. Our results clearly show duplications of both rhabdomeric and ciliary opsins within Tardigrada, forming clades specific to both the Heterotardigrada and Eutardigrada in addition to multiple independent duplications within genera. However, lack of co-temporal, ontogenetic, expression suggests that while tardigrades possess multiple opsins, they are unlikely to be able to distinguish color.

Significance

Opsins are a family of proteins that are involved in a variety of photosensitive tasks, including vision. The evolution of opsins inside arthropods is well understood, but there are little data on the evolution of opsins in closely related clades, such as the tardigrades.

In this article, we introduce a number of new tardigrade opsin sequences, from a range of tardigrade genera that allows us to better understand the evolution of vision within the tardigrades and conjecture on the visual ecology of the clade.

## Introduction

Tardigrada is a microscopic ecdysozoan phylum most famous for their extreme resilience (Nelson et al. 2009; Møbjerg et al. 2011; Hashimoto et al. 2016). They can be found across freshwater, marine, and terrestrial environments, on every continent on earth (Nelson et al. 2009). Despite this disparity, genomic sampling within Tardigrada remains limited, and few complete tardigrade genomes have been sequenced (Hashimoto et al. 2016, Koutsovoulos et al. 2016, Yoshida et al. 2017). Within the Tardigrada, too, there are biases in sampling. The phylum is composed primarily of two subphyla: Eutardigrada and Heterotardigrada (alongside the dubious, monotypic Mesotardigrada; Fleming and Arakawa 2021). These two groups can then be subdivided: the Heterotardigrada into the potentially paraphyletic Arthrotardigrada and the Echinisoidea, and the Eutardigrada into the Apochela and Parachela, both defined based on their claw morphology (Fleming and Arakawa 2021). Unfortunately, the bulk of tardigrade genetic sampling is from within the Apochela, which can be further subvidided into the Macrobiotidae and the Hypsibiidae, meaning that the vast majority of tardigrade diversity has yet to be properly sampled (Hashimoto et al. 2016; Koutsovoulos et al. 2016; Yoshida et al. 2017; Guil et al. 2019; Fleming and Arakawa 2021).

The opsins are photosensitive proteins expressed across Metazoa, in organs as diverse as the eyes, gonads, and brain, where they regulate a number of different light receptive processes (Terakita 2005; Schumann et al. 2016; Fleming et al. 2018; Picciani et al. 2018). Opsins can primarily be divided into three different families: the rhabdomeric opsins (r-opsin), the ciliary opsins (c-opsin), and the group 4 opsins (Feuda et al. 2012, 2014; Schnitzler et al. 2012; Ramirez et al. 2016, Fleming et al. 2020). In the Ecdysozoa, the r-opsins are involved in both visual and nonvisual processes, whilst the ciliary and group 4 opsins are primarily involved in nonvisual processes (Eriksson et al. 2013; Hering and Mayer 2014; Tsukamoto 2014).

Prior research on tardigrade opsins suggested that the Tardigrada possessed only a single rhabdomeric opsin, three ciliary opsins, and one neuropsin (Hering and Mayer 2014); however, more recent analyses suggest that multiple independent duplications of rhabdomeric opsins occurred in Tardigrada (Fleming et al. 2018). This opens the possibility that, similarly to Arthropoda, Tardigrada might be able to distinguish colors. However, poor taxon sampling did not allow testing whether these duplications were specific to Tardigrada as a whole or to specific tardigrade lineages (Fleming et al. 2018).

Differential expression analyses (Anders and Huber 2010) use a combination of genomic and transcriptomic data to take “snapshots” of the expression level of a gene at multiple stages of life, or under different life conditions. Previous differential expression analyses in Tardigrada have explored the effect of the tun state on the expression of heat-sensitive proteins (Yoshida et al. 2017, 2019). By comparing the transcriptomic expression of opsins across tardigrade life stages, we can assess whether different light-sensitive proteins are employed to tackle different ecological challenges within the organism’s life cycle (Cronin and Jinks 2001).

The discovery of multiple rhabdomeric opsins in particular begs the question of their purpose in the context of the tardigrade’s visual ecology. rhabdomeric opsins are primarily associated with visual processes in Protostomia (Hering and Mayer 2014), and duplications within this clade allowed the evolution of color vision in Arthropoda (e.g., Fleming et al. 2018). The presence of duplicated r-opsins in the tardigrade genome suggests the possibility of a more complex role for tardigrade vision than previously thought, including the possibility that Tardigrada might be able to distinguish colors. Previous morphological analyses of tardigrade vision have yet to uncover evidence of color receptivity, as in Arthropoda (Greven 2007; Persson et al. 2012; Fleming et al. 2018). In addition, within the opsin clade generally referred to as “arthropod/ecdysozoan visual opsins,” opsins with nonvisual functions, such as Rh7 (Hering and Mayer 2014; Ni et al. 2017; Sakai et al. 2017; Fleming et al. 2018) exist, and thus the potential that tardigrade r-opsins exist that perform a nonvisual function cannot be ruled out. The presence of multiple r-opsins is intriguing, and differential expression analysis should allow us to test whether these opsins are co-temporally expressed, which is the minimum requirement necessary for an organism to be able to use multiple opsin genes to distinguish colors, as arthropods and vertebrates do (Kelber and Osorio 2010).

Here, we greatly expand the sampling of tardigrade opsin diversity to assess the taxonomic level at which tardigrade opsin duplications occurred. Finally, we use two tardigrade transcriptomes with deep coverage (*Hypsibius exemplaris* and *Ramazzottius variornatus*) to perform a differential expression analysis on their opsins and explore the implications of differential opsin-expression across tardigrade ontogeny.

## Results and Discussion

**Fig. 1.**
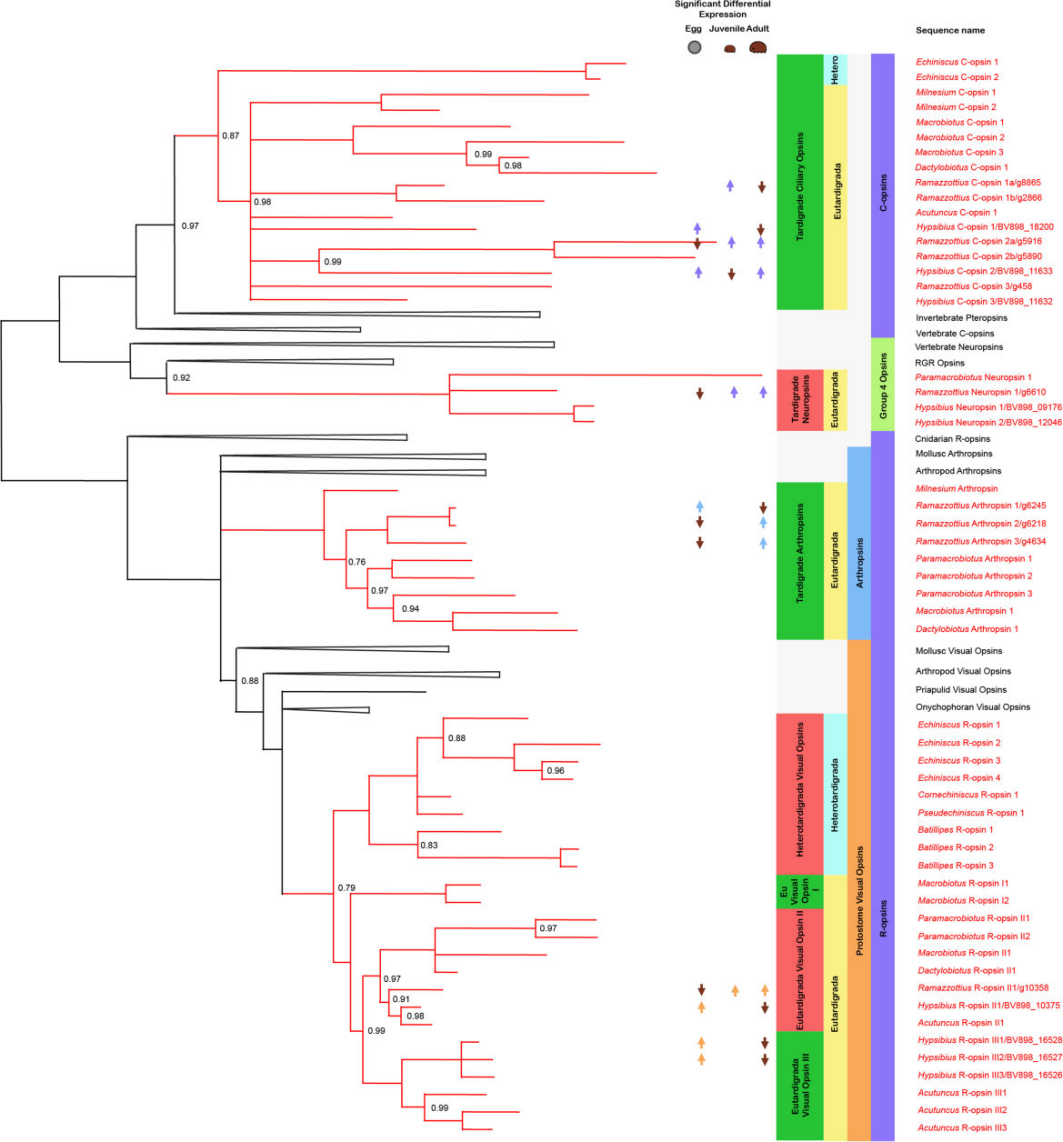
A whole opsin phylogeny, with a specific focus on the tardigrade opsins. New opsins were uncovered in both the Hetero and Eutardigrada, across the ciliary, group 4, and rhabdomeric clades. Posterior probability values for nodes with a posterior probability below 1 are reported, and nodes with a posterior probability below 0.7 have been reduced to polytomies. Red branches represent tardigrade opsins. Significant differential expression of the opsin in a given life stage is also marked on the topology—an up arrow indicates a significantly higher level of expression, and a down arrow a significantly lower level.

Our tardigrade opsin data set consisted of 17 c-opsins, 34 r-opsins, and 4 group 4 opsin homologs. Similarly to Feuda et al. (2012, 2014) and Fleming et al. (2020), we found ciliary and group 4 opsins to constitute the monophyletic sister group of the rhabdomeric opsins (fig. 1, supplementary figs. 1, 2, Supplementary Material online, and supplementary table 1, Supplementary Material online). In *Echiniscus*, we found two ciliary opsins (*Echiniscus* c-opsin 1 and 2), and four rhabdomeric opsins (*Echiniscus* r-opsin 1, 2, 3 and 4). In *Cornechiniscus* and *Pseudechiniscus*, we found only one rhabdomeric opsin each (*Cornechiniscus* r-opsin 1 and *Pseudechiniscus* r-opsin 1). In *Batillipes*, we found three rhabdomeric opsins (*Batillipes* r-opsin 1, 2, and 3). In *Milnesium*, we found two ciliary opsins (*Milnesium* c-opsin 1 and 2) and one rhabdomeric opsin (*Milnesium* arthropsin). In *Macrobiotus*, we found three ciliary opsins (*Macrobiotus* c-opsin 1, 2, and 3) and four rhabdomeric opsins (*Macrobiotus* arthropsin and *Macrobiotus* r-opsin I1, I2, and II1). In *Paramacrobiotus*, we found one neuropsin (*Paramacrobiotus* neuropsin) and five rhabdomeric opsins (*Paramacrobiotus* arthropsins 1, 2, and 3 and *Paramacrobiotus* r-opsin II1 and II2). In *Dactylobiotus*, we found one ciliary opsin (*Dactylobiotus* c-opsin 1) and two rhabdomeric opsins (*Dactylobiotus* arthropsin and *Dactylobiotus* r-opsin II1). In *Acutuncus*, we found one ciliary opsin (*Acutuncus* c-opsin 1) and four rhabdomeric opsins (*Acutuncus* r-opsin II1, III1, III2, and III3). In *Hypsibius*, we found three ciliary opsins (*Hypsibius* c-opsin 1, 2, and 3), two neuropsins (*Hypsibius* neuropsin 1 and 2), and four rhabdomeric opsins (*Hypsibius* r-opsin II1, III1, III2, and III3). Finally, in *Ramazzottius*, we found five ciliary opsins (*Ramazzottius* c-opsin 1a, 1b, 2a, 2b, and 3), one neuropsin (*Ramazzottius* neuropsin), and four rhabdomeric opsins (*Ramazzottius* arthropsin 1, 2, and 3 and *Ramazzottius* r-opsin II1).

Within the ciliary opsins, tardigrade sequences are recovered as sister to the arthropod sequences—the pteropsins. This protostome ciliary opsin group emerges as the sister of the vertebrate pinopsins, as in Fleming et al. (2020). The tardigrade ciliary opsin group is not well resolved, but contains members from both Eutardigrada and Heterotardigrada. The heterotardigrade ciliary opsins resolve as sisters to the eutardigrade opsins, where one independent duplication within *Echiniscus* is observed. Further studies will help elucidate whether this duplication is specific to the genus, or to Heterotardigrada or Echiniscoidea as a group.

The Eutardigrades resolve largely in a polytomy, but four distinct groups can be observed. ciliary Opsins belonging to Macrobiotidae are all more closely related to one another than to any sequences within Hypsibiidae that indicate two independent duplications within the clade (fig. 1). Most notably within this phylogeny, only one ciliary opsin could be found in *Dactylobiotus* and none in *Paramacrobiotus*, suggesting that both may have independently lost these macrobiotid ciliary opsins, or that they were not recovered through lack of sampling.

Within Hypsibiidae, the previously identified *Hypsibius* c-opsins 1, 2, and 3 (Hering and Mayer 2014) are each grouped into specific clades, suggesting at least two independent duplications within Hypsibiidae prior to the divergence of *Acutuncus* and *Ramazzottius* from the other members of the group. Two further independent duplications were observed within *Ramazzottius*.

Within the group 4 opsins, only one new sequence was found, in *Paramacrobiotus*. We were unable to recover any neuropsins from other members of the Macrobiotiidae. However, as neuropsins could not be located within the Heterotardigrada or Apochela, but have previously been located in *Hypsibius*, this may be an issue of sampling, rather than representative of true loss.

### Rhabdomeric Opsins

**Fig. 2.**
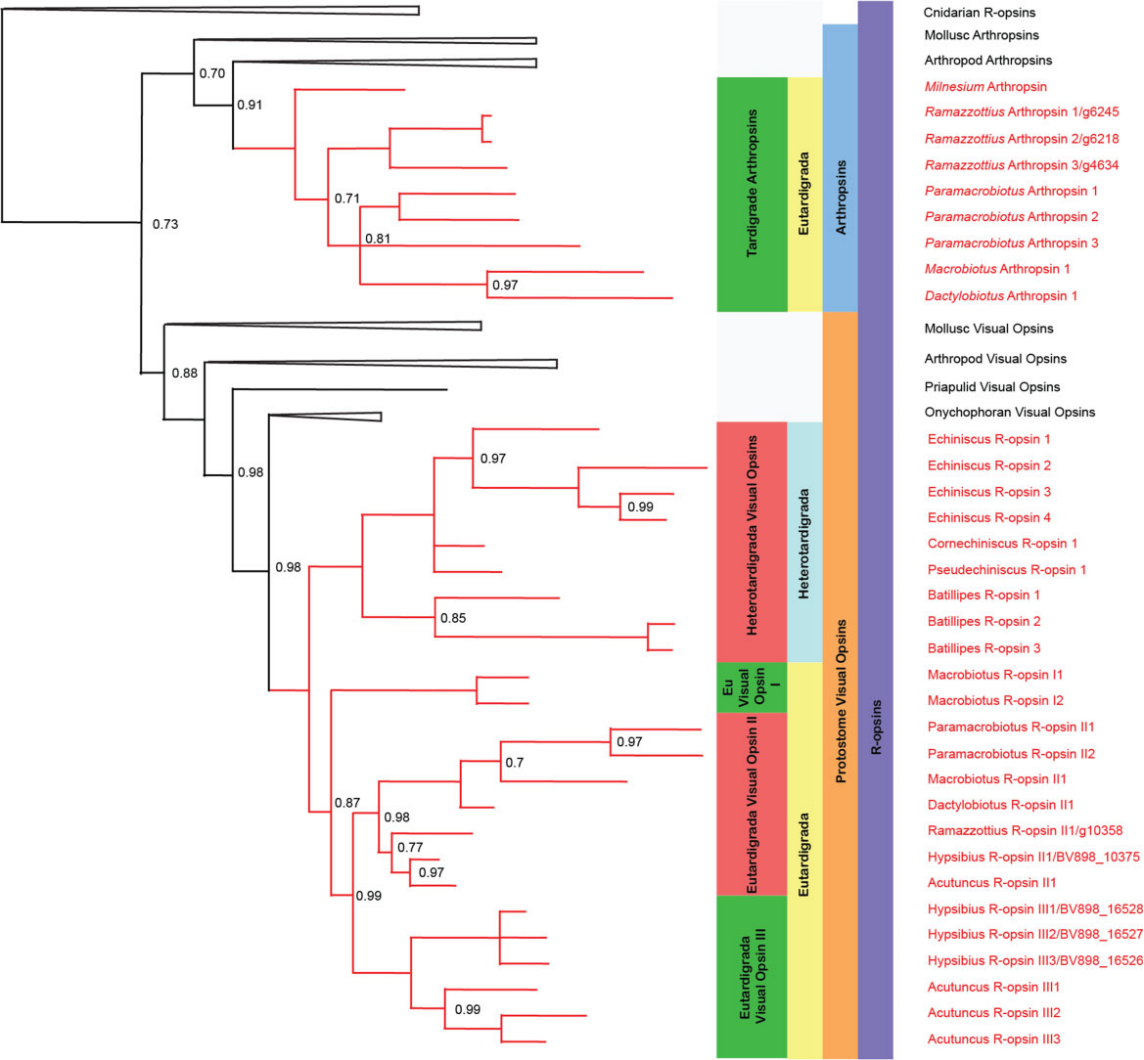
A phylogeny depicting the tardigrade visual opsins in our study. Posterior probability values for nodes with a posterior probability below 1 are reported, and nodes with a posterior probability below 0.7 have been reduced to polytomies.

Our rhabdomeric opsin analysis agrees with previously established rhabdomeric opsin topologies (Hering and Mayer 2014; Fleming et al. 2018). Increased sampling from the Tardigrada, however, clarifies the picture of tardigrade rhabdomeric opsin evolution (fig. 2, supplementary figs. 1 and 2, Supplementary Material online).

We find the first known arthropsins from within Tardigrada—from *Macrobiotus*, *Dactylobiotus*, *Paramacrobiotus, Ramazzottius*, and *Milnesium*. Multiple arthropsin sequences were found in both *Paramacrobiotus areolatus* and *R.variornatus*. These sequences appear to show signs of independent duplication events restricted to the genus in both cases. The function of arthropsin has been speculated upon at length (Eriksson et al. 2013; Schumann et al. 2016; Collantes-Alegre et al. 2018), but is currently unclear. However, arthropsin duplications have only previously been observed within *Daphnia pulex* (Hering and Mayer 2014). Further study of the independent duplications of arthropsin within both Tardigrada and *Daphnia* could advance our understanding of the function of this recently discovered homolog.

Within the tardigrade visual opsins, a pattern of specific gene duplications at multiple systematic levels emerges due to increased sampling in comparison to previous studies. Supporting prior studies, opsin duplications in Tardigrada are present in both Eutardigrada and Heterotardigrada, but occur after the divergence of the two groups from one another (Fleming et al. 2018).

The earliest tardigrade opsin duplication appears to occur within Eutardigrada, following the separation of the Apochela (Apotardigrada) from the Parachela (fig. 2). This duplication forms the “Eutardigrada visual opsin I,” “Eutardigrada visual opsin II,” and “Eutardigrada visual opsin III” groups (fig. 1). Eutardigrada visual opsin I undergoes subsequent duplications within *Macrobiotus* but appears to have been lost in non-macrobiotid Eutardigrada: understanding the nature of this subsequent duplication and general loss will require further sampling from across Eutardigrada, though none of the paramacrobiotid samples in our study possessed sequences belonging to the Eutardigrada visual opsin I group, suggesting the possibility of independent losses within the clade.

Only a single visual opsin was located in *Ramazzottius*, despite multiple separate genome samples being analyzed (fig. 2 and supplementary table 1, Supplementary Material online). However, only *R.variornatus* was examined in our study set—this species lacks eye spots, which are found elsewhere within the genus (Dastych 1993). It may be that more visual opsins are present in species of *Ramazzottius* which possess eyespots, such as *Ramazzottius theroni* (Dastych 1993). Meanwhile, no *Milnesium* visual opsins could be recovered in our data set, despite this being a species that possesses eyespots. This may have been an issue with expression, collection, sequencing or assembling, or may represent a divergent role for tardigrade arthropsin. The authors believe the prior scenario to be more likely, as high-coverage *Milnesium* genomes have not yet been assembled.

Eutardigrada visual opsin III appears to have been lost in the Macrobiotiidae. Although this could be due to poor resolution in the phylogeny, none of our analyses recovered Eutardigrada visual opsins I and III as a single monophyletic group to the exclusion of Eutardigrada visual opsin II. Eutardigrada visual opsin III does, however, show multiple independent duplications in both *Hypsibius* and *Acutuncus*. This suggests that the history of gains and losses within the group may be quite complex.

Within the Heterotardigrada, genus-level duplications can be observed within *Echiniscus* (to the exclusion of other members of the Echiniscoidea) and *Batillipes*. Whether the duplications in *Batillipes* are shared among other members of the Arthrotardigrada is unclear; however, as only *Batillipes* was sampled in this study. In addition, the partial sequences we recovered from these tardigrade visual opsin groups lack the critical Bovine E90 tuning site. This site determines the presence of UV receptivity in insect rhabdomeric opsins (Battelle et al. 2015), and as such the likely wavelength receptivity of these new clades cannot be established without further in vivo study.

### The History of Opsin Duplications in the Tardigrada

The results of this study present a far more complex history of the opsin protein family within the Tardigrada than first thought, with multiple independent duplications and losses characterizing the evolution of the clade (fig. 1).

The common ancestor of the extant Tardigrada appears to have possessed four opsins: one protostome visual opsin, arthropsin, ciliary opsin, and neuropsin. The first opsin duplication within Tardigrada occurred following the divergence of the common ancestor of the Macrobiotiidae and Hypsibiidae, within the ciliary opsins. Further duplications of the ciliary opsins, specific to Parachela, occurred later (fig. 1).

In the protostome visual opsins, one duplication occurred before the divergence of Apochela from Parachela, and further duplications occurred before the divergence of Macrobiotiidae and Hypsibiidae. One copy of protostome visual opsin was then lost in the common ancestor of Macrobiotiidae, reducing the complement to two, before separate independent duplications occurred within those families to obtain the present opsin complements of the clades (fig. 1).

### Differential Expression of Opsins within *Hypsibius* and *Ramazzottius*

We measured the differential expression of nine opsin sequences found in the genome of *H.exemplaris* (four rhabdomeric opsins, three ciliary opsins, and two neuropsins) and ten opsin sequences found in the genome of *R.variornatus* (five ciliary opsins, four rhabdomeric opsins, and one neuropsin; supplementary tables 2–4, Supplementary Material online). Of these 19 opsin sequences, seven of the ten *Ramazzottius* sequences and five of the nine *Hypsibius* sequences were significantly (*q* value < 0.05) differentially expressed between the egg, juvenile, and adult stages, as expressed by a likelihood ratio test.

**Fig. 3.**
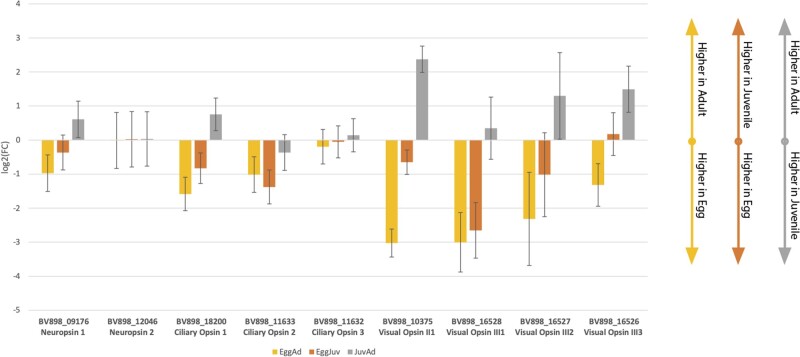
Column graph displaying the comparison of log2 fold changes between each of three comparative conditions of the *Hypsibius* transcriptomes: Egg versus Adult, Egg versus Juvenile, and Juvenile versus Adult. The direction of the column represents the direction of expression of the second condition relative to the first.

Of the nine opsin sequences in the genome of *Hypsibius*, five were found to be significantly differentially expressed, two ciliary opsins, and three rrhabdomeric opsins. Within the ciliary opsins, “BV898_11633,” *Hypsibius* c-opsin 2, showed significantly greater expression in the adult stage, than during the juvenile life stage (adult tpm = 1.08/juvenile tpm = 0.43, *q* value = 0.01, supplementary table 3, Supplementary Material online and fig. 3). The other ciliary opsin, “BV898_18200,” *Hypsibius* c-opsin 1, showed significantly higher expression in the egg stage than in the adult stage (egg tpm = 1.68/adult tpm = 0.96, *q* value = 3e^−3^; supplementary table 3, Supplementary Material online and fig. 3).

Within the rhabdomeric opsins, all three opsins—BV898_10375, BV898_16527, and BV898_16528—show significantly higher expression in the egg stage when compared with the adult life stage (egg tpm = 12.78, 2.78, 0.18/adult tpm = 0.06, 0.34, 0.05 and *q* values = 5e^−12^, 7e^−4^, and 1e^−3^ respectively; supplementary table 3, Supplementary Material online and fig. 3). All of these rhabdomeric opsins correspond to the tardigrade visual opsin clade.

However, total tpm across all life stages for all of these sequences, with the exception of BV898_10375, was low (tpm < 10), and for BV898_16527 and BV898_16528, tpm did not exceed 0.5 in any of the examined states. As such, these results in particular must be inferred with caution, and they suggest that these opsin sequences, though present in the *Hypsibius* genome are not highly expressed at all—this is true of all nine opsin sequences involved in this study, regardless of whether they were significantly differentially expressed. This could be because the number of cells expressing these proteins was limited within the whole body transcriptome, and as such, these values are diluted (Yoshida et al. 2017).

**Fig. 4.**
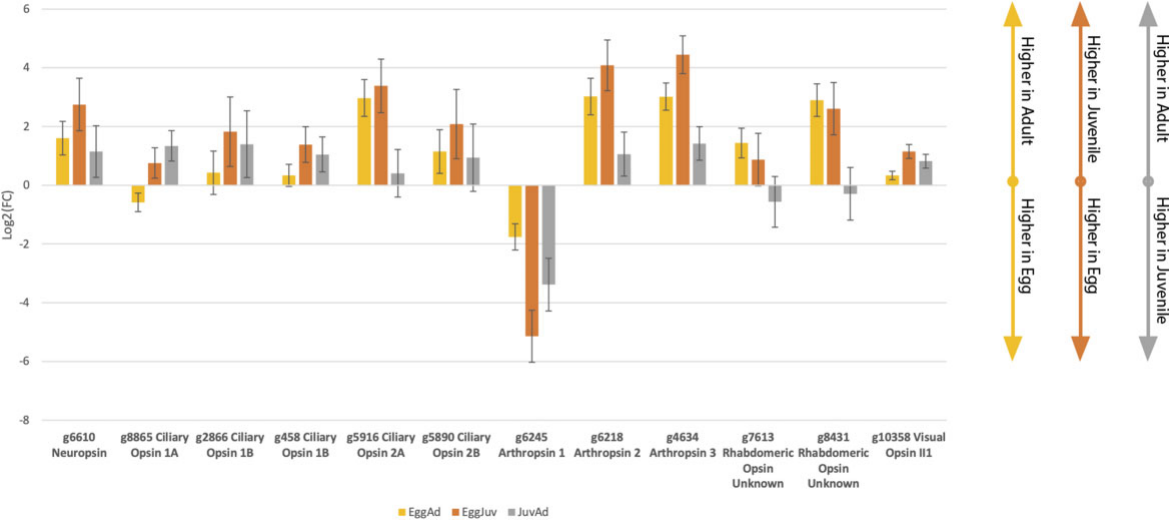
Column graph displaying the comparison of log2 fold changes between each of three comparative conditions of the *Ramazzottius* transcriptomes: Egg versus Adult, Egg versus Juvenile, and Juvenile versus Adult. The direction of the column represents the direction of expression of the second condition relative to the first.

Within *Ramazzottius*, seven of the 14 opsin sequences were found to be significantly differentially expressed, two ciliary opsins, four rhabdomeric opsins, and neuropsin. Of the two ciliary opsins that are differentially expressed, g5916 and g8865, g5916 is more significantly expressed in the Adult when compared with the egg stage (adult tpm = 1.70/egg tpm = 0.17, *q* value = 7.12e^−6^; supplementary table 4, Supplementary Material online and fig. 4). The second ciliary opsin, however, g8865, is significantly more expressed in the juvenile stage when compared with the adult stage (juvenile tpm = 4.48/adult tpm = 2.22, *q* value = 0.04; supplementary table 4, Supplementary Material online and fig. 4), but not when the juvenile stage is compared with egg stage (juvenile tpm = 4.48/egg tpm = 3.18, *q* value = 0.23; supplementary table 3, Supplementary Material online and fig. 4) or when the egg stage is compared with the adult stage (egg tpm = 3.18/adult tpm = 2.22, *q* value = 0.10; supplementary table 4, Supplementary Material online and fig. 4), suggesting that this opsin is more highly expressed during the growth stages of Tardigrada.

The four rhabdomeric opsins in *Ramazzottius* display a very different expression pattern when compared with the rhabdomeric opsins of *Hypsibius*, as only a single rhabdomeric opsin, g6245, shows significantly greater expression in the egg stage when compared with the adult stage (adult tpm = 3.45/egg tpm = 9.39, *q* value = 2e^−4^; supplementary table 4, Supplementary Material online and fig. 4). The remaining five significantly differentially expressed rhabdomeric opsins show significantly higher expression in the adult stage when compared with egg stage (supplementary table 4, Supplementary Material online and fig. 4). The significantly differentially expressed neuropsin is also more highly expressed in both the free-living stages of *Hypsibius* (adult tpm = 2.10/egg tpm = 0.85 *q* value = 9e^−3^, juvenile tpm = 3.87/egg tpm = 0.85 *q* value = 6e^−3^, Juvenile/Egg; supplementary table 4, Supplementary Material online and fig. 4). g6245 also resolves as an independently duplicated *Ramazzottius* arthropsin: as *Ramazzottius* has undergone two independent duplications within the arthropsin clade, this too may be an adaptation towards a specialized egg arthropsin.

When compared with the phylogeny we can see that expression patterns within the ciliary opsins are conserved between *Hypsibius* and *Ramazzottius*. *Ramazzottius* g5916 is a member of the same clade as *Hypsibius* c-opsin 2, and *Ramazzottius* g885 is a member of the same clade as *Hypsibius* c-opsin 1 (fig. 1), and both of these pairs possess similar expression patterns. As the former is more highly expressed in the juvenile, and the latter in the adult stage, it may be that this represents two opsins evolving to fulfil differing, but complementary, functions following a shared duplication event. In addition, both of these opsins appear key to the free-living state of the tardigrade, a mode which is not observed in the r-opsins. This suggests that differences in the photoecology of juvenile and adult tardigrades have driven this opsin divergence within Tardigrada. Prior research in the Tardigrada has also found differential expression of the cryptobiotic response proteins CAHS and SAHS through ontology (Yoshida et al. 2019). These orthologs are instrumental in the maintenance of tardigrade desiccation cryptobiosis at all of its life stages, and it is possible that the differential expression of opsin is also linked to this response, considering the common linkage between light intensity and aridity (Yoshida et al. 2019). Notably, *Hypsibius* c-opsin 3 showed no signs of differential expression across the organism’s ontogeny.

Within the rhabdomeric opsins, however, a different pattern is observed. Both BV_10375 and g10358 are homologous members of Eutardigrada visual opsin family 2 (fig. 1). BV_10375 is significantly more highly expressed in the egg as opposed to the adult, whereas g10358 is more highly expressed in the adult as opposed to the egg. The other rhabdomeric opsins that displayed significant differences in expression in *H.exemplaris*, BV_16527 and BV_16528, do not possess a *Ramazzottius* homolog, and are additionally also more highly expressed within the egg stages. Meanwhile, in *Ramazzottius*, g6245 is an opsin sequence with no clear homolog in *Hypsibius*, as an arthropsin (fig. 1). It appears to be significantly more highly expressed during the egg stage of the organism.

As vision is not present in the egg, this recurrent high expression in the egg stage could indicate that these rhabdomeric opsin genes are employed for nonvisual functions important in the development or timing of photosensitivity, but further work would be required to determine the exact function of these new tardigrade egg rhabdomeric opsins. Considering their increased expresssion within the egg stage of the Tardigrada, these opsins may not be visual in nature, despite being rhabdomeric opsins. Within the arthropods, nonvisual rhabdomeric opsins have been found to exist within the Rh7 clade, and as such there exists a possibility of a similar event occurring independently within the Tardigrada that cannot be dismissed (Ni et al. 2017; Sakai et al. 2017).

However, despite tardigrade’s multiple visual opsins, we find it unlikely that they are capable of color vision in the same fashion as the Arthropoda. This would require expression of multiple opsins within the eyespot and the ability to distinguish between the varying responses of each opsin pigment to light. Further analysis of the Tardigrada would be required to positive confirm the lack of dual expression within the eye.

Not all opsins showed signs of significant differential expression. Three *Hypsibius* opsins—*Hypsibius* c-opsin 3, visual opsin III-3 and neuropsin—were all consistently expressed across all stages. Three opsins in *Ramazzottius—*c-opsin 1b, c-opsin 2b, and neuropsin—also showed consistent expression across all stages (supplementary tables 3 and 4, Supplementary Material online). Within neuropsin, it appears that this expression pattern is species-specific. However, in the ciliary opsins, c-opsin 2 is more heavily expressed in the adult, whereas c-opsin 1 is less expressed in the same stage, and this pattern is conserved between *Ramazzottius* and *Hypsibius*, perhaps suggesting a shared origin.

## Conclusion

Tardigrade opsins have undergone a number of duplications independent to clades within the phylum, and are differentially expressed across the ontogeny of the organism, with some being more highly expressed within the egg. This suggests that light-sensitive processes within the egg stage of Tardigrada are particularly important, and that the natural history of opsins within the phylum is much more complex than previously thought, and intrinsically bound to the history of the habitats that they have inhabited. Though color vision is unlikely to be possessed by the clade, multiple opsins suggest a wider array of light-sensitive behaviors than previously thought. Tardigrade ciliary opsins in particular change expression between the juvenile and adult stage, suggesting that these sequences may play an important role in the ontogeny of members of the group, and the different ecological roles they fulfil at different stages within their lives.

## Materials and Methods

### Identification of New Opsins

New opsin genes were identified using BLAST (*e* value = 10e-7) (Camacho et al. 2009) against a set of opsin query sequences—the data set of Hering and Mayer (2014), alongside a selection of 2 previously published tardigrade genomes and transcriptomes, and 33 new genomes and transcriptomes sequenced by the authors (supplementary table 1, Supplementary Material online). New genomes were sequenced using the protocol outlined in Arakawa et al. (2016). Sequences with a significant hit were assessed in Prosite (De Castro et al. 2006), and only sequences with a Retinal Binding Domain were retained. This procedure allowed the identification of 89 tardigrade opsins. When accounting for cases of multiple sampling from the same species, we recovered 17 ciliary, 34 rhabdomeric, and 4 group 4 unique opsin homologs.

### Phylogenetic Analyses

#### Broad Classification of Tardigrade Opsins

New opsin sequences were aligned alongside the Fleming et al. (2020) “minimal opsin” data set using MUSCLE 3.8 (Edgar 2004). This tree provides an even sampling across the three major opsin families, and as such should provide a valuable guide to identify the phylogenetic affinity of the individual tardigrade opsins.

This phylogenetic tree was constructed using the GTR+G model in Phylobayes 4.1 (Lartillot and Philippe 2004, 2006; Lartillot et al. 2007). This model has previously been shown to be appropriate for opsin studies (Feuda et al. 2014). Convergence was assessed by comparing the maximum discrepancies observed over the bipartitions and effective sample size in bpcomp (maxdiff: 0.288, meandiff: 0.012) and tracecomp. For all analyses two independent chains were run. A burnin of 50% of the sample size was used for all analyses, sampling every 50th tree following the burnin period.

From the results of this analysis (supplementary fig. 1, Supplementary Material online), sequences were then assigned identities corresponding to the r-opsin, c-opsin, and group 4 families.

### Differential Expression of Opsins in *H. exemplaris* and *R. variornatus*

Two existing deep coverage genomes (BioProject PRJNA 369262) of the eutardigrades *H.exemplaris* and *R.variornatus*, alongside available mRNA transcriptome data from eggs, juveniles, and adults of the same species were used to perform a differential expression analysis of opsins in Tardigrada. We used all of the ontogeny-related transcriptomes generated within the Bioproject. Within the aforementioned bioproject, three SRR replicates were deposited for each day of the development cycle of both species, from the first day of the egg cycle. The methods used to sequence this data are detailed in Yoshida et al. (2017). We grouped the transcripts into three categories based on their descriptions within the SRA Run Selector for the Bioproject: Egg, Juvenile, and Adult. This resulted in 15 egg stage transcripts and 12 adult stage transcripts for both *Hypsibius* and *Ramazzottius*, and 21 and 3 juvenile stage transcripts for *Hypsibius* and *Ramazzottius*, respectively. The transcriptomic data have been released to the sequence read archive (https://www.ncbi.nlm.nih.gov/sra) with accessions SRR5218203 to SRR5218250 (see also supplementary table 2, Supplementary Material online).

These mRNA-Seq reads were aligned to the relevant species and gene expression (measured in TPM—transcripts per million) was then calculated using Kallisto v0.46.1 (Bray et al. 2016). Sequences were mapped using BWA 0.7.17 (Li and Durbin 2010) and, after summarizing the read count of each gene, DESeq2 3.11 (Love et al. 2014) was used to normalize the read counts and then make the differential expression calculations. *q* values greater than 0.05 were considered significant, and variations in TPM are discussed more directly for each significant result in the relevant section. The effective length, estimated fragment counts with and without bias correction (est_count and eff_count), alongside the log-fold standard error (lfcSE) and adjusted *P* values (*q* values, identified as adj*P*) and unadjusted *P* values (*P*) are available in our Supplementary Material online.

### Phylogenetic Analyses within the Rhabdomeric Opsin Family

Following the division of the new opsin sequences into r-opsins, c-opsins, and group 4 opsins, a further data set was constructed, including only the 79 new tardigrade visual r-opsins and well-characterized rhabdomeric opsins gathered from NCBI, previously published in (Fleming et al. 2020). By using more well-characterized rhabdomeric opsins, we were able to obtain a better understanding of the position of the new sequences with reference to other members of this diverse group, and proceed with identification of their potential ecological role. The data set was aligned in MUSCLE 3.8 (Edgar 2004), and a preliminary phylogenetic tree was constructed and assessed using PhyML 3.0 (Guindon et al. 2010) and the WAG model (Whelan and Goldman 2001). A final tree was constructed using the GTR+G model in Phylobayes 4.1, as reported above (maxdiff: 0.171, meandiff: 0.008).

## Supplementary Material

Supplementary data are available at *Genome Biology and Evolution* online.

## Supplementary Material

evab164_Supplementary_Data
