# Supplementary material for: New Tardigrade Opsins and Differential Expression Analyses Show Ontogenic Variation in Light Perception
Source: Genome Biol Evol. 2021 Jul 13;13(8):evab164. doi: 10.1093/gbe/evab164 (PMC8350360; doi:10.1093/gbe/evab164)
Supplement: evab164_Supplementary_Data [file evab164_supplementary_data.zip › SupplementalInformation_Jun29.tar!/SupplementalInformation_Jun29.zip!/SupplementalInformation_Jun29/SupplementaryFigures_Jun29/SupplementaryFigure1.pdf]

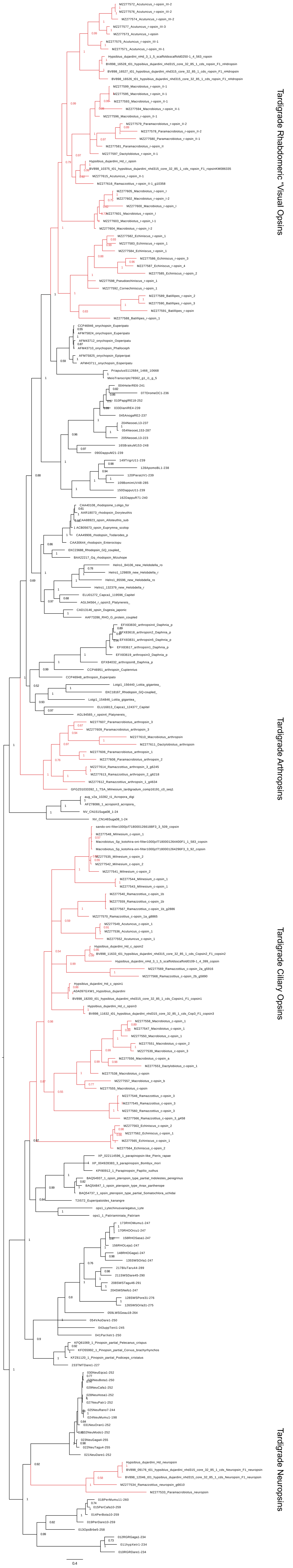
Tardigrade Rhabdomeric "Visual" Opsins
Tardigrade Arthropsins
Tardigrade Ciliary Opsins
Tardigrade Neuropsins
0.4
