# Supplementary material for: New Tardigrade Opsins and Differential Expression Analyses Show Ontogenic Variation in Light Perception
Source: Genome Biol Evol. 2021 Jul 13;13(8):evab164. doi: 10.1093/gbe/evab164 (PMC8350360; doi:10.1093/gbe/evab164)
Supplement: evab164_Supplementary_Data [file evab164_supplementary_data.zip › SupplementalInformation_Jun29.tar!/SupplementalInformation_Jun29.zip!/SupplementalInformation_Jun29/SupplementaryFigures_Jun29/SupplementaryFigure2.pdf]

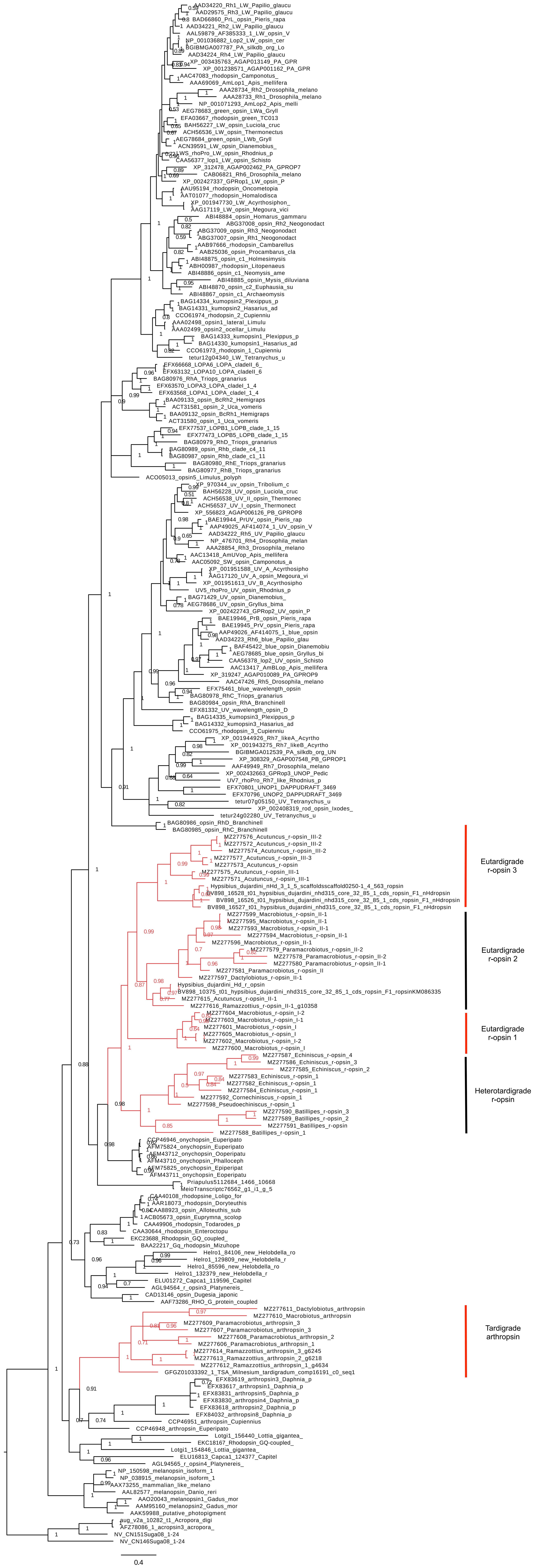

AAD34220_Rh1_LW_Papilio_glaucu
AAD29575_Rh3_LW_Papilio_glaucu
BAD66860_PrL_opsin_Pieris_rapa
AAD34221_Rh2_LW_Papilio_glaucu
AAL59879_AF385333_1_LW_opsin_V
NP_001036882_Lop2_LW_opsin_cer
BGIBMGA007787_PA_silkdb_org_Lo
AAD34224_Rh4_LW_Papilio_glaucu
XP_003435763_AGAP013149_PA_GPR
XP_001238571_AGAP001162_PA_GPR
AAC47083_rhodopsin_Camponotus_
AAA69069_AmLop1_Apis_mellifera
AAA28734_Rh2_Drosophila_melano
AAA28733_Rh1_Drosophila_melano
NP_001071293_AmLop2_Apis_melli
AEG78683_green_opsin_LWa_Gryll
EFA03667_rhodopsin_green_TC013
BAH56227_LW_opsin_Luciola_cruc
ACH56536_LW_opsin_Thermonectus
AEG78684_green_opsin_LWb_Gryll
ACN39591_LW_opsin_Dianemobius_
LWS_rhoPro_LW_opsin_Rhodnius_p
CAA56377_lop1_LW_opsin_Schisto
XP_312478_AGAP002462_PA_GPROP7
CAB06821_Rh6_Drosophila_melano
XP_002427337_GPRop1_LW_opsin_P
AAU95194_rhodopsin_Oncometopia
AAT01077_rhodopsin_Homalodisca
XP_001947730_LW_Acyrthosiphon_
AAG17119_LW_opsin_Megoura_vici
ABI48884_opsin_Homarus_gammaru
ABG37008_opsin_Rh2_Neogonodact
ABG37009_opsin_Rh3_Neogonodact
ABG37007_opsin_Rh1_Neogonodact
AAB97666_rhodopsin_Cambarellus
AAB25036_opsin_Procambarus_cla
ABI48875_opsin_c1_Holmesimysis
ABH00987_rhodopsin_Litopenaeus
ABI48886_opsin_c1_Neomysis_ame
ABI48885_opsin_Mysis_diluviana
ABI48870_opsin_c2_Euphausia_su
ABI48867_opsin_c1_Archaeomysis
BAG14334_kumopsin2_Plexippus_p
BAG14331_kumopsin2_Hasarius_ad
CCO61974_rhodopsin_2_Cupienniu
AAA02498_opsin1_lateral_Limulu
AAA02499_opsin2_ocellar_Limulu
BAG14333_kumopsin1_Plexippus_p
BAG14330_kumopsin1_Hasarius_ad
CCO61973_rhodopsin_1_Cupienniu
tetur12g04340_LW_Tetranychus_u
EFX66668_LOPA6_LOPA_cladeI_6_
EFX63132_LOPA10_LOPA_cladeI_6
BAG80976_RhA_Triops_granarius
EFX63570_LOPA3_LOPA_cladel_1_4
EFX63568_LOPA1_LOPA_cladel_1_4
BAA09133_opsin_BcRh2_Hemigraps
ACT31581_opsin_2_Uca_vomeris
BAA09132_opsin_BcRh1_Hemigraps
ACT31580_opsin_1_Uca_vomeris
EFX77537_LOPB1_LOPB_clade_1_15
EFX77473_LOPB5_LOPB_clade_1_15
BAG80979_RhD_Triops_granarius
BAG80989_opsin_Rhb_clade_c4_11
BAG80987_opsin_Rhb_clade_c1_11
BAG80980_RhE_Triops_granarius
BAG80977_RhB_Triops_granarius
ACO05013_opsin5_Limulus_polyph
XP_970344_uv_opsin_Tribolium_c
BAH56228_UV_opsin_Luciola_cruc
ACH56538_UV_II_opsin_Thermonec
ACH56537_UV_I_opsin_Thermonect
XP_556823_AGAP006126_PB_GPROP8
BAE19944_PrUV_opsin_Pieris_rap
AAP49025_AF414074_1_UV_opsin_V
AAD34222_Rh5_UV_Papilio_glaucu
NP_476701_Rh4_Drosophila_melan
AAA28854_Rh3_Drosophila_melano
AAC13418_AmUVop_Apis_mellifera
AAC05092_SW_opsin_Camponotus_a
XP_001951588_UV_A_Acyrthosipho
AAG17120_UV_A_opsin_Megoura_vi
XP_001951613_UV_B_Acyrthosipho
UV5_rhoPro_UV_opsin_Rhodnius_p
BAG71429_UV_opsin_Dianemobius_
AEG78686_UV_opsin_Gryllus_bima
XP_002422743_GPRop2_UV_opsin_P
BAE19946_PrB_opsin_Pieris_rapa
BAE19945_PrV_opsin_Pieris_rapa
AAP49026_AF414075_1_blue_opsin
AAD34223_Rh6_blue_Papilio_glau
BAF45422_blue_opsin_Dianemobiu
AEG78685_blue_opsin_Gryllus_bi
CAA56378_lop2_UV_opsin_Schisto
AAC13417_AmBLop_Apis_mellifera
XP_319247_AGAP010089_PA_GPROP9
AAC47426_Rh5_Drosophila_melano
EFX75461_blue_wavelength_opsin
BAG80978_RhC_Triops_granarius
BAG80984_opsin_RhA_Branchinell
EFX81332_UV_wavelength_opsin_D
BAG14335_kumopsin3_Plexippus_p
BAG14332_kumopsin3_Hasarius_ad
CCO61975_rhodopsin_3_Cupienniu
XP_001944926_Rh7_likeA_Acyrtho
XP_001943275_Rh7_likeB_Acyrtho
BGIBMGA012539_PA_silkdb_org_UN
XP_308329_AGAP007548_PB_GPROP1
AAF49949_Rh7_Drosophila_melano
XP_002432663_GPRop3_UNOP_Pedic
UV7_rhoPro_Rh7_like_Rhodnius_p
EFX70801_UNOP1_DAPPUDRAFT_3469
EFX70796_UNOP2_DAPPUDRAFT_3469
tetur07g05150_UV_Tetranychus_u
XP_002408319_rod_opsin_Ixodes_
tetur24g02280_UV_Tetranychus_u
BAG80986_opsin_RhD_Branchinell
BAG80985_opsin_RhC_Branchinell
MZ277576_Acutuncus_r-opsin_III-2
MZ277572_Acutuncus_r-opsin_III-2
MZ277574_Acutuncus_r-opsin_III-2
MZ277577_Acutuncus_r-opsin_III-3
MZ277573_Acutuncus_r-opsin
MZ277575_Acutuncus_r-opsin_III-1
MZ277571_Acutuncus_r-opsin_III-1
Hypsibius_dujardini_nHd_3_1_5_scaffoldsscaffold0250-1_4_563_ropsin
BV898_16528_t01_hypsibius_dujardini_nhd315_core_32_85_1_cds_ropsin_F1_nHdropsin
BV898_16526_t01_hypsibius_dujardini_nhd315_core_32_85_1_cds_ropsin_F1_nHdropsin
BV898_16527_t01_hypsibius_dujardini_nhd315_core_32_85_1_cds_ropsin_F1_nHdropsin
Eutardigrade r-opsin 3
MZ277599_Macrobiotus_r-opsin_II-1
MZ277595_Macrobiotus_r-opsin_II-1
MZ277593_Macrobiotus_r-opsin_II-1
MZ277594_Macrobiotus_r-opsin_II-1
MZ277596_Macrobiotus_r-opsin_II-1
MZ277579_Paramacrobiotus_r-opsin_II-2
MZ277578_Paramacrobiotus_r-opsin_II-2
MZ277580_Paramacrobiotus_r-opsin_II-1
MZ277581_Paramacrobiotus_r-opsin_II
MZ277597_Dactylobiotus_r-opsin_II-1
Hypsibius_dujardini_Hd_r_opsin
BV898_10375_t01_hypsibius_dujardini_nhd315_core_32_85_1_cds_ropsin_F1_ropsinKM086335
MZ277615_Acutuncus_r-opsin_II-1
MZ277616_Ramazzottius_r-opsin_II-1_g10358
Eutardigrade r-opsin 2
MZ277604_Macrobiotus_r-opsin_I-2
MZ277603_Macrobiotus_r-opsin_I-1
MZ277601_Macrobiotus_r-opsin_I
MZ277605_Macrobiotus_r-opsin_I
MZ277602_Macrobiotus_r-opsin_I-2
MZ277600_Macrobiotus_r-opsin_I
Eutardigrade r-opsin 1
MZ277587_Echiniscus_r-opsin_4
MZ277586_Echiniscus_r-opsin_3
MZ277585_Echiniscus_r-opsin_2
MZ277583_Echiniscus_r-opsin_1
MZ277582_Echiniscus_r-opsin_1
MZ277584_Echiniscus_r-opsin_1
MZ277592_Cornechiniscus_r-opsin_1
MZ277598_Pseudoechiniscus_r-opsin_1
MZ277590_Batillipes_r-opsin_3
MZ277589_Batillipes_r-opsin_2
MZ277591_Batillipes_r-opsin
MZ277588_Batillipes_r-opsin_1
Heterotardigrade r-opsin
CCP46946_onychopsin_Euperipato
AFM75824_onychopsin_Euperipato
AFM43712_onychopsin_Ooperipatu
AFM43710_onychopsin_Phalloceph
AFM75825_onychopsin_Epiperipat
AFM43711_onychopsin_Eoperipatu
Priapulus5112684_1466_10668
MeioTranscriptc76562_g1_i1_g_5
CAA40108_rhodopsine_Loligo_for
AAR18073_rhodopsin_Doryteuthis
CAA88923_opsin_Alloteuthis_sub
ACB05673_opsin_Euprymna_scolop
CAA49906_rhodopsin_Todarodes_p
CAA30644_rhodopsin_Enteroctopu
EKC23688_Rhodopsin_GQ_coupled_
BAA22217_Gq_rhodopsin_Mizuhope
Helro1_84106_new_Helobdella_ro
Helro1_129809_new_Helobdella_r
Helro1_85596_new_Helobdella_ro
Helro1_132379_new_Helobdella_r
ELU01272_Capca1_119596_Capitel
AGL94564_r_opsin3_Platynereis_
CAD13146_opsin_Dugesia_japonic
AAF73286_RHO_G_protein_coupled
MZ277611_Dactylobiotus_arthropsin
MZ277610_Macrobiotus_arthropsin
MZ277609_Paramacrobiotus_arthropsin_3
MZ277607_Paramacrobiotus_arthropsin_3
MZ277608_Paramacrobiotus_arthropsin_2
MZ277606_Paramacrobiotus_arthropsin_1
MZ277614_Ramazzottius_arthropsin_3_g6245
MZ277613_Ramazzottius_arthropsin_2_g6218
MZ277612_Ramazzottius_arthropsin_1_g4634
GFGZ01033392_1_TSA_Milnesium_tardigradum_comp16191_c0_seq1
Tardigrade arthropsin
EFX83619_arthropsin3_Daphnia_p
EFX83617_arthropsin1_Daphnia_p
EFX83831_arthropsin5_Daphnia_p
EFX83830_arthropsin4_Daphnia_p
EFX83618_arthropsin2_Daphnia_p
EFX84032_arthropsin8_Daphnia_p
CCP46951_arthropsin_Cupiennius
CCP46948_arthropsin_Euperipato
Lotgi1_156440_Lottia_gigantea_
EKC18167_Rhodopsin_GQ-coupled_
Lotgi1_154846_Lottia_gigantea_
ELU16813_Capca1_124377_Capitel
AGL94565_r_opsin4_Platynereis_
NP_150598_melanopsin_isoform_1
NP_038915_melanopsin_isoform_1
AAX73255_mammalian_like_melano
AAL82577_melanopsin_Danio_reri
AAO20043_melanopsin1_Gadus_mor
AAM95160_melanopsin2_Gadus_mor
AAK59988_putative_photopigment
aug_v2a_10282_t1_Acropora_digi
AFZ78086_1_acropsin3_acropora_
NV_CN151Suga08_1-24
NV_CN146Suga08_1-24
0.4
